# Composition, uniqueness and connectivity across tropical coastal lagoon habitats in the Red Sea

**DOI:** 10.1186/s12898-020-00329-z

**Published:** 2020-11-23

**Authors:** Zahra Alsaffar, João Cúrdia, Xabier Irigoien, Susana Carvalho

**Affiliations:** 1 Red Sea Research Centre, King Abdullah University of Science and Technology (KAUST), Thuwal, 23955-6900 Saudi Arabia; 2 Chemistry Department, College of Science, King Saud University (KSU), Riyadh, P.O. Box 2455, 11451 Saudi Arabia; 3 AZTI - Marine Research, Herrera Kaia, Pasaia, 20100 Spain; 4 IKERBASQUE, Basque Foundation for Science, Bilbao, 48013 Spain

**Keywords:** Biodiversity, Inter-annual variability, Spatial variability, Macrobenthic communities, Tropical habitats, Seascape connectivity, Red Sea

## Abstract

**Background:**

Tropical habitats and their associated environmental characteristics play a critical role in shaping macroinvertebrate communities. Assessing patterns of diversity over space and time and investigating the factors that control and generate those patterns is critical for conservation efforts. However, these factors are still poorly understood in sub-tropical and tropical regions. The present study applied a combination of uni- and multivariate techniques to test whether patterns of biodiversity, composition, and structure of macrobenthic assemblages change across different lagoon habitats (two mangrove sites; two seagrass meadows with varying levels of vegetation cover; and an unvegetated subtidal area) and between seasons and years.

**Results:**

In total, 4771 invertebrates were identified belonging to 272 operational taxonomic units (OTUs). We observed that macrobenthic lagoon assemblages are diverse, heterogeneous and that the most evident biological pattern was spatial rather than temporal. To investigate whether macrofaunal patterns within the lagoon habitats (mangrove, seagrass, unvegetated area) changed through the time, we analysed each habitat separately. The results showed high seasonal and inter-annual variability in the macrofaunal patterns. However, the seagrass beds that are characterized by variable vegetation cover, through time, showed comparatively higher stability (with the lowest values of inter-annual variability and a high number of resident taxa). These results support the theory that seagrass habitat complexity promotes diversity and density of macrobenthic assemblages. Despite the structural and functional importance of seagrass beds documented in this study, the results also highlighted the small-scale heterogeneity of tropical habitats that may serve as biodiversity repositories.

**Conclusions:**

Comprehensive approaches at the “seascape” level are required for improved ecosystem management and to maintain connectivity patterns amongst habitats. This is particularly true along the Saudi Arabian coast of the Red Sea, which is currently experiencing rapid coastal development. Also, considering the high temporal variability (seasonal and inter-annual) of tropical shallow-water habitats, monitoring and management plans must include temporal scales.

## Background

Coastal lagoons are important transition systems providing essential socio-economic goods and services (e.g. shore protection, fisheries, carbon sequestration) [1–3]. Coastal lagoons harbour well-adapted and sometimes unique assemblages of species, which play a vital role directly supporting local populations. These ecosystems are naturally stressed on daily to annual-time scales [4–8] and display high environmental variability (e.g. temperature, salinity, primary productivity, nutrients, dissolved oxygen). Such variability is reflected in the biological patterns that alter in response to the new environmental conditions. Lagoon ecosystems are also being increasingly affected by human disturbances that can compromise their ecological and socio-economic values [5, 9–12].

Subtropical and tropical coastal lagoons encompass a range of essential soft-substrate habitats, such as mangroves, seagrasses and unvegetated bottoms. These habitats are associated with different environmental conditions, resulting not only from their location along the depth profile but also their structural complexity, and biological assemblages [13–16]. However, while these habitats contain a diverse range of organisms spatial distribution patterns and connectivity in subtropical and tropical lagoon habitats have mainly been assessed using fish and other mobile marine fauna [17–23]. Studies describing and comparing macrobenthic distribution patterns and the strength of connectivity linkages across different shallow-water tropical lagoon habitats are particularly limited compared to temperate systems (e.g. [15, 24–27]). Spatial differences in the community can provide information regarding the ecological requirements of species. For example, species able to colonize multiple habitats will most likely be less sensitive to environmental changes, whereas those more directly associated with a specific habitat may be less tolerant to environmental changes. In general, harsher environmental conditions are observed in the intertidal area, dominated by mangrove trees, with conditions being attenuated with increasing depth, a pattern that is associated with a consistent increase in species richness and abundance [28, 29]. Indeed, mangrove habitats are characterized as unfavourable environments influenced by high salinity, high fluctuation of temperature, desiccation, and poor soil condition (depleted oxygen) [30]. On the other hand, if undisturbed, seagrass habitats provide comparatively more stable environmental conditions through time [31–33] as well as protection from predators [34].

Furthermore, the knowledge about the role of temporal variability in driving macrobenthic patterns is still scarce [35–41]. While seasonal changes in tropical regions are comparatively less distinct than in temperate regions [42], temporal variability in benthic patterns exists [39, 43, 44]. Investigating temporal variability patterns is essential to obtain a deeper knowledge of the dynamics and processes regulating lagoon communities. Indeed, considering the current scenario of global climate change, it is critical to better understand how the distribution patterns of organisms in these habitats are changing and particularly how they respond to changes in temperature and other key environmental drivers [1, 45]. Temporal variation patterns in the abundance and composition of macrofaunal invertebrates have been intensively studied in temperate coastal ecosystems in relation to environmental variables [46–49]. Temporal variability in temperature and food availability, for example, can influence recruitment events with consequences for the structure, distribution, and abundance of the community [50–52]. Similarly, sediment composition, organic matter, and vegetation cover, which may vary in time, are also main drivers of observed ecological patterns. However, most of those studies have been conducted in temperate regions and, more recently in polar habitats (e.g. [53–57]). Comparatively, less attention has been dedicated to sub-tropical and tropical areas (e.g. [58–61]). This is even more striking in regards to the assessment of inter-annual variability (but see [40, 62, 63]).

Assuming that harsher environmental conditions will occur towards the intertidal area (i.e. mangrove habitats), we hypothesise (i) a decrease in species richness (i.e. the total number of species) and in the number of exclusive species from subtidal to intertidal areas, as less resistant species are progressively excluded along the the environmental gradient. We also hypothesise that (ii) shallow water seagrass meadows will harbour higher numbers of species particularly compared with unvegetated bottoms, as a result of habitat complexity, protection from predators and food availability [64–66]. Likewise, we hypothesise (iii) that temporal changes will be less evident in subtidal (vegetated and unvegetated) than intertidal habitats [30, 67] and that subtidal seagrasses areas will support more stable communities through time. Ecologically related management decisions require a sound knowledge of the biodiversity of the ecosystem. By assessing the variability in spatial and temporal patterns of macro benthic organisms we expand on the existing knowledge on tropical coastal lagoons which are sensitive as well as ecologically and economically valuable.

## Results

### Macrobenthic community composition: general characterization and connectivity among habitats

**Fig. 1:**
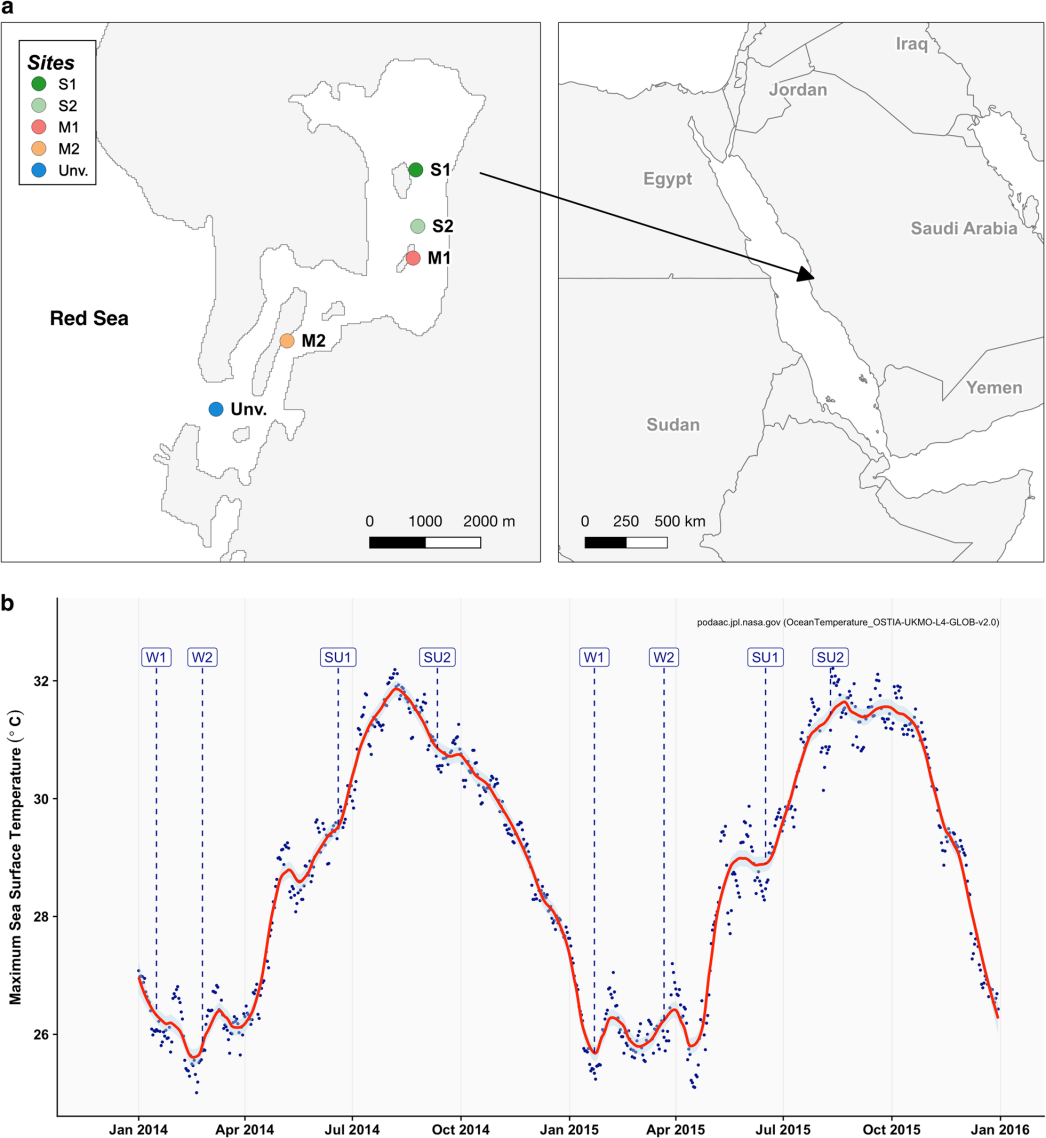
**a** Map showing the locations of the habitats in the lagoon. **b** Annual variability in sea surface temperature in the lagoon during the study period. M1 and M2, mangrove; S1 and S2, seagrass; and unvegetated area (Unv.). SU1 and SU2, summer sampling dates 1 and 2; W1 and W2, winter sampling dates 1 and 2. The map was produced by the authors using data freely available (http://www.thematicmapping.org/downloads/world_borders.php; https://www.gadm.org/download_country_v3.html, Saudi Arabia)

A total of 4771 invertebrates were identified within the different habitats surveyed in the lagoon (Fig. 1a), belonging to 272 operational taxonomic units (OTUs) distributed among 11 phyla, 16 classes, 40 orders, and 80 families. Annelida dominated both in abundance and number of taxa, contributing to, respectively, 51.0% and 42.0% of the total values. Sipuncula (15.0%), Arthropoda (13.0%), Mollusca (12.0%), and Echinodermata (7.0%) also contributed to the overall density. Regarding the number of species, Arthropoda (28.0%) and Mollusca (18.0%) were, along with Annelida, the phyla contributing the most to the total number of species.

At the species level, the sipunculid *Phascolion (Phascolion) strombus strombus* (12.2% of the total abundance) was the most abundant species, followed by the polychaetes *Simplisetia erythraeensis* (5.8%), *Eunice indica* (4.4%), *Ceratocephale* sp. (3.3%), *Aonides* sp. (2.7%), *Lumbrineris* sp.1 (2.7%), and *Lysidice unicornis* (2.6%), the amphipod *Metaprotella africana* (3.3%), and the bivalves *Barbatia foliata5* (2.7%) and *Paphies angusta* (2.4%). Most of these taxa were found in at least four of the studied sites, except for *Metaprotella africana* (exclusive to S1) and *Barbatia foliata*, exclusive to seagrass habitats (S1 and S2). All the remaining taxa contributed to less than 2% of the total abundance.

Only eight taxa (3% the total number of taxa) spanned across the five habitats. Most of them were polychaetes (*Capitellethus* sp., *Drillonereis* sp., *Euclymene* spp., *Lumbrineris* sp.1, *Lysidice unicornis*, *Notomastus* spp.). Nemertea (und.) and the sipunculid *Phascolion (Phascolion) strombus strombus* were also observed across the five sites. *Simplisetia erythraeensis* was absent from the unvegetated site. There were 62 taxa shared between intertidal and subtidal sites, and only 18 exclusive species to the mangrove habitats (as a whole), representing 6.6% of the of the gamma diversity (2.2%, M2; 4.4%, M1). On the other hand, subtidal habitats showed a rather consistent percentage of exclusive species, ranging from 29.4% in S1, 32.3% in S2 and 33.8% in the unvegetated area (S1: 12.8%; S2: 18.4%; Unvegetated: 15.1% of the gamma diversity, i.e. the total number of taxa observed in the lagoon).

Both seagrass habitats showed a higher percentage of resident species (i.e. species present in over 85% of the sampling dates in a certain habitat) compared to mangrove and unvegetated areas (Table 2). In terms of the number of individuals, those taxa contributed to 45.0% and 34.0% for S1 and S2, respectively, of the site’s total abundance. S2 showed a more balanced distribution of the four habitat preference traits analysed (i.e. resident, frequent, occasional, rare) and relatively stable numbers throughout the study period (Table 2). Regardless of the habitat, occasional species accounted for more than 12.6% of the total number of species.

**Fig. 2:**
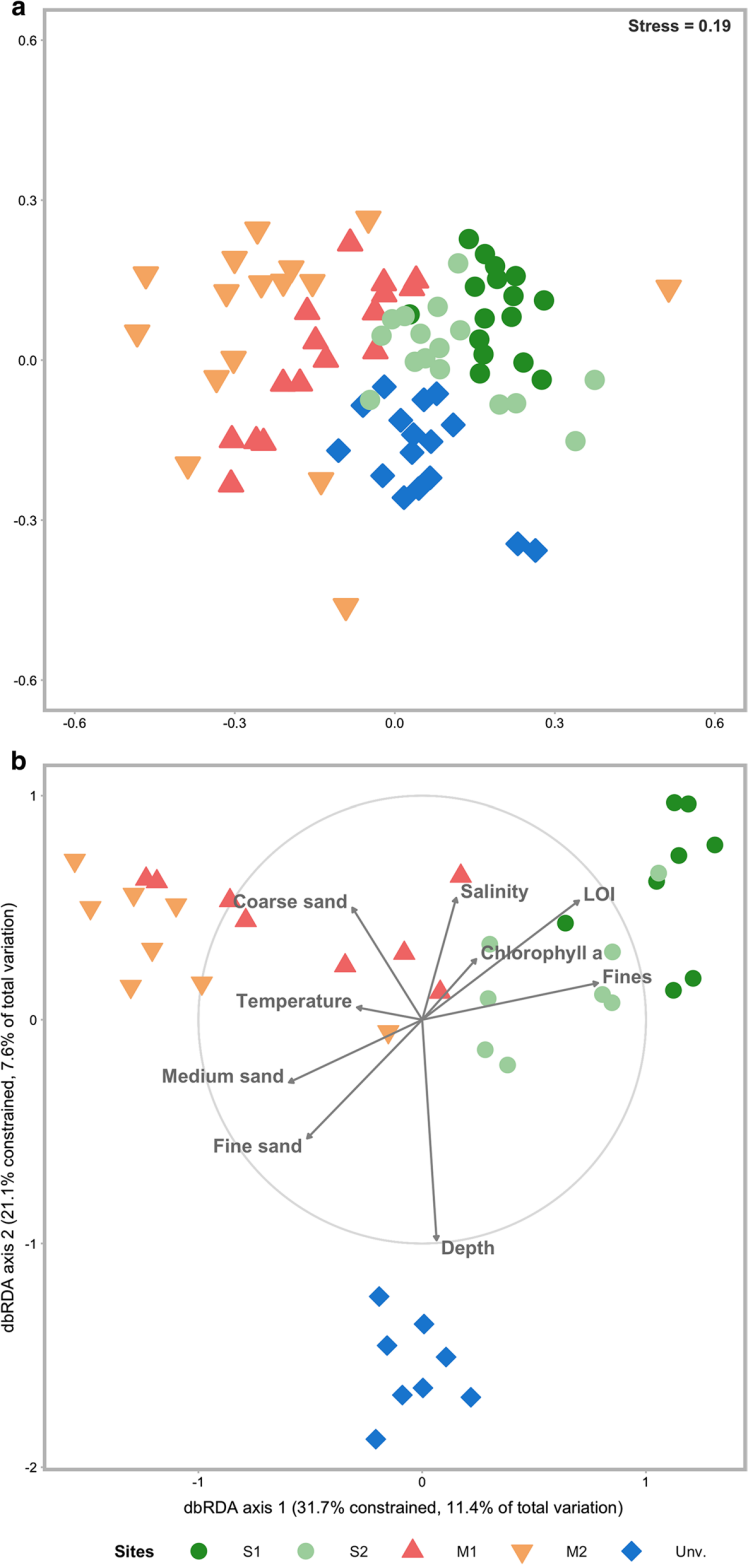
Multivariate analysis of the community data. **a** Ordination (non-metric multivariate dimensional analysis) and classification diagram of the sampling habitats based on the Bray–Curtis dissimilarity on non-transformed data. **b** Distance-based redundancy analysis (dbRDA) plot based on a set of environmental variables; salinity, temperature, depth, grain size fractions (coarse sand medium sand, fine sand, fines), organic matter: LOI (%) and chlorophyll *a* on biological data from lagoon habitats; M1 and M2, mangrove; S1 and S2, seagrass; and unvegetated area (Unv). The points represented the sampling events (winter 1, winter 2, summer 1, and summer 2) for 2014 and 2015. Coarse sand and fines data are square root transformed and LOI log_e_ transformed. Length and direction of vectors indicate the strength and direction of the relationship

Macrobenthic patterns of variability across the lagoon seascape show that the community was structured by habitat with limited seascape ecological connectivity across the different habitats (Fig. 2a). The environmental data gathered partially explained the multivariate variability of the biological data with the two first axes of the distance-based redundancy analysis (dbRDA) explaining more than half of the constrained variability but only 19.1% of the total variability of the biological communities. The dbRDA plot reinforces a clear separation of the communities inhabiting mangrove areas, S1, and the unvegetated habitat, whereas S2 presented affinities (i.e. higher connectivity) with either S1 or mangrove stations depending on the sampling period (Fig. 2b). Samples from the unvegetated habitat were associated with depth and percentages of medium and fine sand. Seagrass habitats (particularly S1) were separated based on the higher silt and clay (fine particles) content, whereas mangrove habitats presented a slightly higher percentage of coarse sand. Multivariate patterns suggest that the nature of the biotope itself drives the composition and structure of macrobenthic communities. The investigation of temporal variability was undertaken for each habitat separately.

### Temporal variability within habitats

**Fig. 3:**
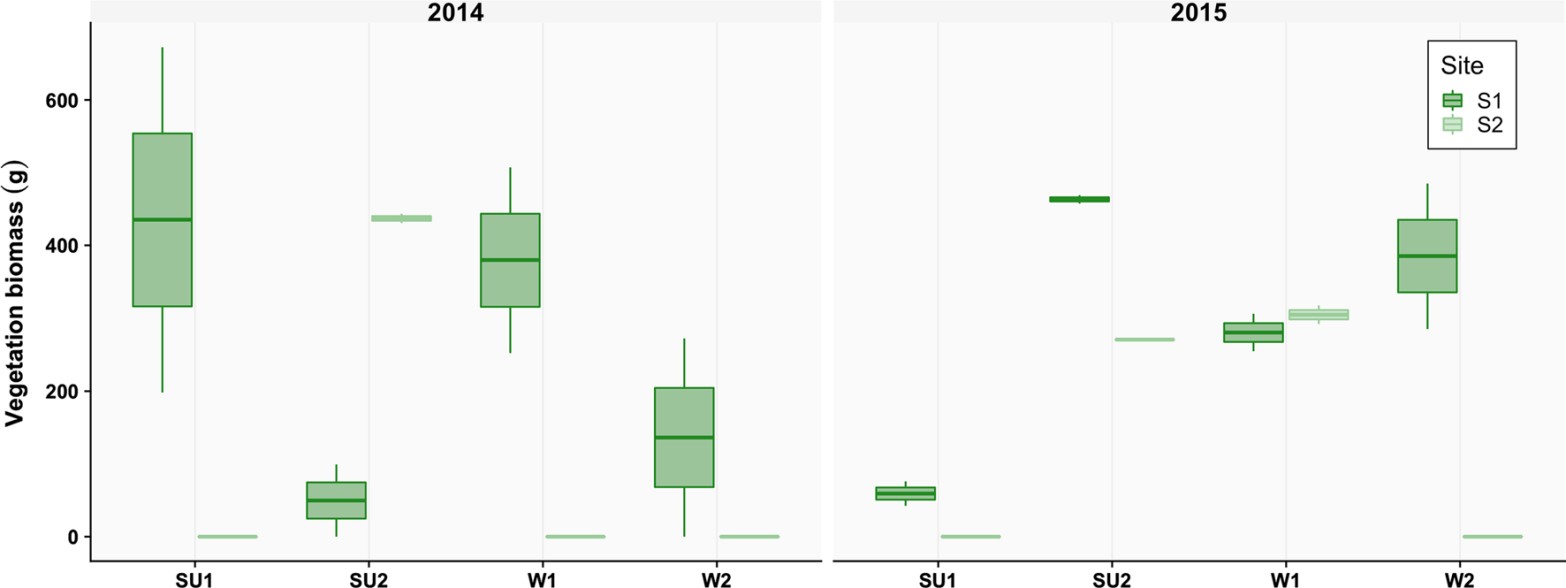
Biomass of seagrass plants along the study period (2014–2015) in both seagrass stations. SU1 and SU2, summer sampling dates 1 and 2; W1 and W2, winter sampling dates 1 and 2. S1 and S2, seagrass sites

The high variability patterns in the seagrass biomass along the study period (Fig. 3) was reflected in the biological changes but was not fully aligned with the temporal pattern in sea water temperature (Fig. 1b). When analysing the full dataset and regardless the diversity metric considered, S2 consistently presented the highest number of taxa (155, observed; 184.8–219.7, estimated), whereas M2 was the poorest taxa site. Density was also higher at S2 (801.9 ind.m^−2^) and lowest at the unvegetated area (388.8 ind.m^−2^) (Table 1).

**Table 1:** Total number of Operational Taxonomic Units (OTUs), estimated number of taxa based on Chao, Jacknife (1st order) and Bootstrap, and average density (ind.m^−2^) per habitat. M1 and M2, mangrove; S1 and S2, seagrass

Habitat	No. OTUs	Chao	Jacknife	Bootstrap	Av. Density (ind.m-^2^)
M1	65	113.0 ± 23.80	95.0 ± 12.92	78.0 ± 6.94	634.4
M2	38	151.9 ± 79.76	63.3 ± 8.51	48.1 ± 3.81	434.4
S1	119	177.8 ± 21.07	171.5 ± 18.13	142.5 ± 9.68	722.5
S2	*155*	*212.2 ± 18.19*	*219.7 ± 18.29*	*184.8 ± 9.36*	*801.9*
Unvegetated	121	156.2 ± 13.81	163.2 ± 14.94	140.9 ± 8.05	388.8

Highest value per metric is presented in italic

**Fig. 4:**
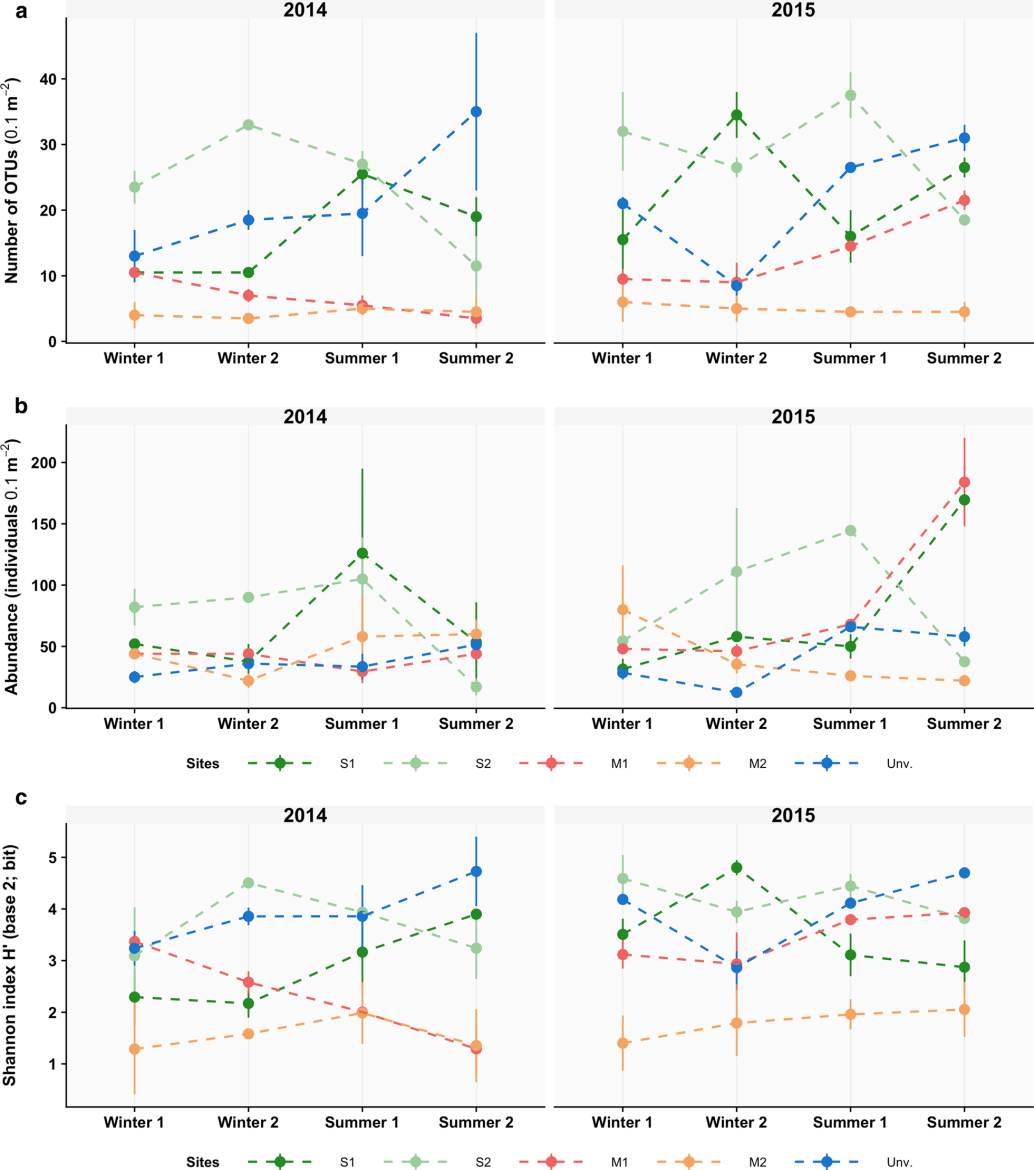
Alpha-diversity metrics per habitat and over time. **a** Number of Operation Taxonomic Units (OTUs), **b** density, and **c** Shannon–Wiener diversity. M1 and M2, mangrove; S1 and S2, seagrass; and unvegetated area (Unv)

In general, a higher number of OTUs were observed in the subtidal habitats than the intertidal mangrove areas (Fig. 4a), with M2 showing a consistently depressed number of taxa across all sampling dates. Abundance was also generally higher within seagrass meadows (Fig. 4b). M2 also presented the lowest Shannon–Wiener diversity whereas, in general, higher values were observed at S2 or at the unvegetated habitat (Fig. 4c).

Biological similarity within each habitat was markedly low, ranging from 14% (M2) to 25% (S1) (Table 2). Both habitats also showed a higher dominance with only four and six species contributing to over 62% of the habitat’s abundance, respectively. In the remaining habitats, a minimum of 13 taxa was needed to reach the same level of abundance (Table 2). Except for S1, where none of the dominant taxa was a polychaete, this group dominated all the other habitats. S1 was dominated by a sipunculid (*Phascolion (Phascolion) strombus strombus*), two bivalves (*Barbatia foliata* and *Cardiolucina semperiana*), one amphipod (*Metaprotella africana*) and two echinoderms (*Aquilonastra burtoni* and *Amphioplus cyrtacanthus*).

**Table 2:** Cumulative percentage of the taxa (Cum %) contributing to more than 60% of each habitat’s total abundance

Taxa	Tax. Group	Cum %	HPT
M1–Overall similarity 18%			
*Ceratocephale* sp.	POL	8.9	OCCA
*Phascolion (Phascolion) strombus strombus*	SIP	16.7	FRE
*Protodorvillea* sp.	POL	24.6	RARE
*Eunice indica*	POL	30.5	FRE
*Diogenes costatus*	DEC	35.3	FRE
*Lysidice unicornis*	POL	38.8	OCCA
*Simplisetia erythraeensis*	POL	42.4	OCCA
*Aspidosiphon* sp.	SIP	45.5	RARE
*Syllis hyllebergi*	POL	48.7	OCCA
*Marphysa macintoshi*	POL	51.7	RES
*Scoletoma* sp.	POL	54.5	OCCA
*Thalamita poissonii*	DEC	57.2	OCCA
*Linopherus* sp.	POL	59.6	RARE
Nemertea	NEM	62.0	OCCA
M2-Overall similarity 14%			
*Simplisetia erythraeensis*	POL	32.1	FRE
*Paphies angusta*	BIV	48.8	RARE
*Ceratocephale* sp.	POL	58.0	OCCA
*Paucibranchia adenensis*	POL	63.1	OCCA
S1-Overall similarity 25%			
*Phascolion (Phascolion) strombus strombus*	SIP	28.3	RES
*Metaprotella africana*	AMP	42.1	RARE
*Barbatia foliata*	BIV	49.6	RES
*Aquilonastra burtoni*	AST	56.5	RES
*Amphioplus cyrtacanthus*	OPH	59.9	FRE
*Cardiolucina semperiana*	BIV	62.2	RES
S2-Overall similarity 19%			
*Phascolion (Phascolion) strombus strombus*	SIP	12.1	RES
*Eunice indica*	POL	20.0	RES
*Lumbrineris* sp1	POL	27.1	RES
*Aonides* sp.	POL	33.5	OCCA
*Lysidice unicornis*	POL	39.1	FRE
*Amphioplus (Lymanella) hastatus*	OPH	43.7	RES
*Barbatia foliata*	BIV	46.9	OCCA
*Paradoneis lyra*	POL	49.9	OCCA
*Euclymene* spp.	POL	52.8	FRE
*Notomastus* sp.	POL	55.3	FRE
*Cardiolucina semperiana*	BIV	57.7	FRE
*Pseudosympodomma persicum*	CUM	59.8	FRE
*Goniada multidentata*	POL	61.6	RES
Unvegetated-Overall similarity 16%			
*Eunice indica*	POL	5.5	FRE
*Aonides* sp.	POL	10.0	SEASONAL
*Lumbrineris* sp2	POL	13.7	OCCA
*Ampelisca brevicornis*	AMP	17.2	FRE
*Ancilla* sp3	GAS	20.3	OCCA
*Glycinde bonhourei*	POL	23.3	OCCA
*Schizaster gibberulus*	ECH	26.2	FRE
*Diplocirrus* sp.	POL	28.9	SEASONAL
*Phascolion (Phascolion) strombus strombus*	SIP	31.7	OCCA
Nemertea	NEM	34.1	OCCA
*Amphioplus cyrtacanthus*	OPH	36.3	RARE
*Leptochela aculeocaudata*	DEC	38.6	OCCA
*Lumbrineris* sp1	POL	40.8	OCCA
*Kirkegaardia* sp1	POL	42.9	SEASONAL
*Lumbrineris* sp3	POL	45.0	RARE
*Paucibranchia adenensis *	POL	47.1	OCCA
*Magelona cincta*	POL	49.0	OCCA
*Macrophthalmus graeffei*	DEC	50.8	SEASONAL
*Amphiodia duplicata*	OPH	52.4	OCCA
*Antalis rossati*	SCA	54.0	OCCA
*Dentalium bisexangulatum*	SCA	55.6	OCCA
*Branchiostoma lanceolatum*	CEPH	57.1	OCCA
*Chaetozone setosa*	POL	58.4	OCCA
*Euclymene* spp	POL	59.6	OCCA
*Streblosoma persica*	POL	60.9	RARE

For each taxa, it is provided the taxonomic group (Tax. Group) and the Habitat Preference Trait (HPT). *Pol* Polychaeta, *SIP* Sipuncula, *BIV* Bivalvia, *DEC* Decapoda, *NEM* Nemertea, *AMP* Amphipoda, *Cum* Cumacea, *OPH* Ophiuroidea, *AST* Asteroidea, *CEPH* Cephalochordata, *ECH* Echinoidea, *SCA* Scaphopoda, *GAS* Gastropoda, *RES* resident, *FRE* frequent, *OCCA* occasional, SEASONAL and RARE. See text for further explanations

**Fig. 5:**
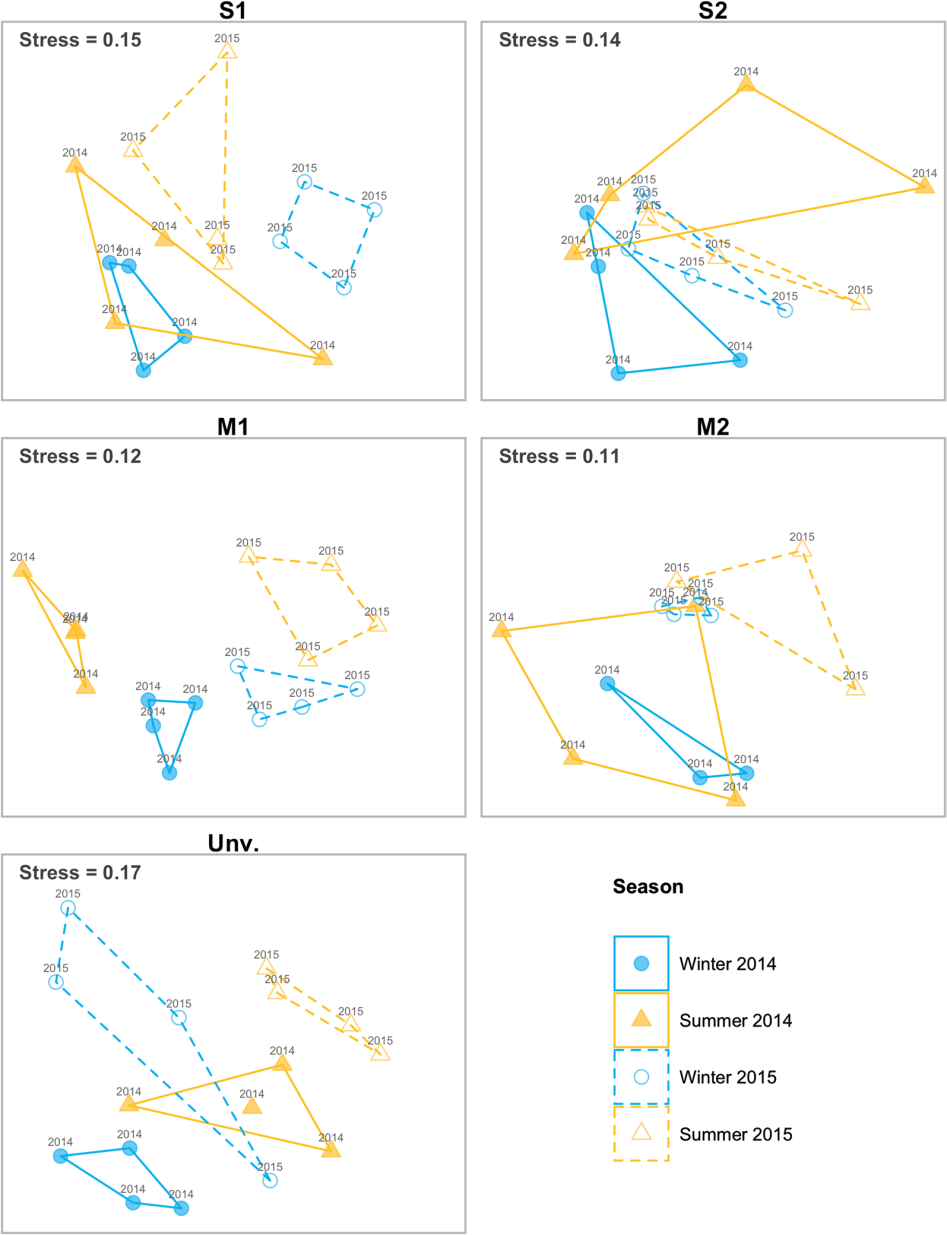
Non-metric multidimensional scaling (nMDS) based on Bray–Curtis dissimilarity matrices based on untransformed data, for temporal variation in the structure of macrobenthic assemblages within each habitat. M1 and M2, mangrove; S1 and S2, seagrass; and unvegetated area (Unv)

Temporal variation in the structure of macrobenthic assemblages within each habitat examined on the basis of the Bray–Curtis and Jaccard resemblance measures indicated different patterns depending on the habitat in analysis. Major differences were not detected between metrics and therefore only plots for Bray–Curtis matrices are presented (Fig. 5). The results of the Permutational Multivariate Analysis of Variance (PERMANOVA) confirmed different temporal trajectories in the analysed habitats (Table 3). Both resemblance metrics applied to M1 and S1 datasets showed a significant interaction of the main factors (Year x Season). The pair-wise tests indicated for M1 a significant inter-annual difference both in winter and summer. For S1, inter-annual differences were only detected in winter. With regard to seasonal differences, S1 presented significant variability in both years (except in the composition–Jaccard-for 2015) but in M1 differences were only detected in 2014 (Table 3). Macrobenthic communities at M2 and S2 showed significant inter-annual variability (except for S2 with presence/absence) (Table 3). Finally, the unvegetated area showed significant and independent seasonal and inter-annual variability (Table 3).

**Table 3:** Two-way PERMANOVA model and pair-wise tests based on Bray–Curtis and Jaccard matrices within habitats among seasons and year (Year and Season interaction; Yr x Se)

Source	Df	MS	Pseudo-F	P(perm)	MS	Pseudo-F	P(perm)
		Bray–Curtis			Jaccard		
M1							
Yr	1	14,075	6.5403	0.001	12,833	4.884	0.001
Se	1	7999.3	3.7171	0.001	7602.5	2.8934	0.001
Yr x Se	1	3967.2	1.8434	0.025	4439.2	1.6895	0.012
Res	12	2152			2627.6		
M2							
Yr	1	8802.9	2.4787	0.007	6405	1.6328	0.041
Se	1	3050.7	0.85902	0.608	4207.3	1.0726	0.36
Yr x Se	1	3968.6	1.1175	0.362	2779.8	0.70866	0.859
Res	12	3551.4			3922.6		
S1							
Yr	1	6362.4	2.71	0.002	5844.2	1.9678	0.002
Se	1	3610.6	1.5379	0.086	5086.2	1.7125	0.009
Yr x Se	1	4735.3	2.0169	0.008	4624.4	1.5571	0.026
Res	12	2347.8			2970		
S2							
Yr	1	5513.2	1.7404	0.038	4583.2	1.3682	0.089
Se	1	3369.6	1.0637	0.376	4300.4	1.2838	0.149
Yr x Se	1	3296.2	1.0405	0.36	4002.9	1.195	0.196
Res	12	3167.9			3349.8		
Unv.							
Yr	1	7397.4	2.7142	0.001	5802.3	1.831	0.003
Se	1	8791.8	3.2258	0.001	8686.2	2.7411	0.001
*Yr x Se*	1	4248.6	1.5589	0.039	4144	1.3077	0.129
Res	12	2725.5			3168.9		
Pair-wise tests							
Term ‘Yr x Se’ for pairs of levels of factor ‘Year’							
Within level ‘Su’ of factor ‘Season’							
M1		Groups	t	P(perm)		t	P(perm)
		2014, 2015	2.2928	0.026		2.0907	0.024
Within level ‘W’ of factor ‘Season’							
M1		2014, 2015	1.7421	0.028		1.5051	0.038
S1		2014, 2015	2.0553	0.023		1.5675	0.022
Term ‘Yr x Se’ for pairs of levels of factor ‘Season’							
Within level ‘2014′ of factor ‘Year’							
M1		Su, W	2.1788	0.03		1.9213	0.029
S1		Su, W	1.2833	0.031		1.4205	0.037
Within level ‘2015’ of factor ‘Year’							
S1		Su, W	1.3824	0.029			

M1 and M2, mangrove; S1 and S2, seagrass; and unvegetated area (Unv)

## Discussion

This study investigated the distribution patterns of macrobenthic communities inhabiting adjacent shallow-water habitats in a tropical coastal lagoon with particular focus on how they are connected and how communities within each habitat vary over time. Even though ecological seascape connectivity has been previously demonstrated particularly for fish, information on the benthic dynamics in tropical lagoons is still scarce. The Al Qadimah lagoon, likewise other tropical lagoons, encompasses a wide range of habitats including both hard (not addressed here) and soft-substrates. Within the latter, changes in the vegetation cover result in a mosaic of habitats with different sedimentary properties that will determine the structure of local macrobenthic communities [68]. Here, we observed a clear zonation of the benthic communities, driven by habitat-related factors acting at varying spatial scales [69]. The present results also provided new insights into the temporal variability (seasonal and inter-annual) of different lagoon shallow-water habitats in a tropical seascape.

### Uniqueness of lagoon habitats within the seascape

A clear pattern of habitat-dependent association was observed with the different habitats harbouring distinct macrobenthic assemblages. The high spatial variability of macrofaunal patterns is most likely linked to the heterogeneity of the seascape and to the high contribution of rare species to the overall abundance. Recent studies showed that biological variability is driven by the relative high contribution of rare and common species, with rare species playing a major role in the temporal patterns, as a result of their vulnerability to fluctuations in environmental conditions (e.g. [70, 71]).

Subtidal habitats harboured 70% of the total number of species. Overall, seagrass habitats showed the highest number of taxa, which agrees with previous studies [65, 68, 72–74]. Variability was, however, high and significant differences within the subtidal area were not detected. The structural complexity provided by the seagrass canopy and the developed rhizome and root systems that contribute to sediment stability may favour the development of diverse communities [70, 75, 76]. In the tropics, the canopy can play an additional critical role providing shade that can attenuate the effects of sea water temperature [8] that in the study region can reach over 32 °C in the summer. Yet, we found that denser seagrass meadows are not always the most favourable habitats for several invertebrates, even though this result may be site-dependent [77–80]. Indeed, the site displaying the highest variability in the cover during the study period, showed the highest number of taxa, density of individuals, and exclusive number of species (32.3% of the site’s total number of species). Dense vegetation can physically obstruct the movement of large burrowing macroinvertebrates [68, 81]. Also, despite the increased aeration within the sediment due to the developed root system [82], the decomposition of the high amounts of organic matter will require increased oxygen consumption and result in anoxic regions and accumulation of toxic products [83, 84]. Therefore, vegetated areas with comparatlively lower cover might harbour higher species numbers as a result of species avoiding toxic anoxic conditions in densely covered areas [85].

Within mangrove habitats species encounter harsh physical environmental conditions (e.g. high salinity, hypoxia, desiccation, high concentration of toxins) and in general nitrogen limitation (C/N ratio often > 100; although mangroves in the Red Sea are carbon limited compared to other locations [86]) due to a low nutritional value of the main source of organic matter, i.e. leaf litter [25]. Under these consitions, populations of a few tolerant/opportunistic species dominate the macrobenthic communities [25, 87]. In the present study, the deepest mangrove area (M2) was dominated by only four species, the polychaetes *Simplisetia erythraeensis*, *Ceratocephale* sp. and *Paucibranchia adenensis*, and the bivalve *Paphies* *angusta* contributed to over 60% of the total abundance. In the shallowest mangrove area, despite the dominance of polychaetes, the sipunculid (*Phascolion (Phascolion) strombus strombus*) and some decapods (*Diogenes costatus* and *Thalamita poissonii*) were also co-dominant. Decapods are critical players for the ecosystem functioning of these habitats by processing leaf litter and oxygenating sediment through their burrows [88, 89] and therefore their dominance in the habitat is not surprising. As observed elsewhere, mangrove habitats showed the lowest number of species compared to nearby seagrass and unvegetated substrates, as previously found [90, 91].

### Connectedness and stability at the scale of the seascape

In the present study, nearby seagrass meadows differed in cover and depth location, which might have resulted in limited similarity in faunal communities (both habitats shared 35.0% of total number of species). Higher similarities (~ higher seascape connectivity) were detected among subtidal habitats than between those and mangroves (intertidal habitats). Nevertheless, 62 taxa, representing 22.8% of the gamma diversity, were shared between intertidal and subtidal habitats, suggesting that several species may utilize contrasting yet adjacent habitats within the lagoon seascape. Despite the fact that the overlap of species across the five habitats is lower (eight taxa; 2.9% of the total number of taxa) than previously reported [92, 93], the present study suggests the connectivity between intertidal and subtidal areas and the need for integrated management measures. The results obtained may result from the low hydrodynamic conditions present but information on the hydrographic patterns is non-existent. The effect of tides can result in displacement of specimens through water movement [94] and depending on their height can also expose organisms to desiccation for variable periods of time, which may hinder the distribution of most of the species toward the intertidal area. Specially, when analysed together, mangrove habitats contributed to 6.6% (M1, 4.4%; M2, 2.2%) of the gamma diversity, contrasting with the unvegetated subtidal area and the seagrass meadows that supported, respectively, 15.1% and 31.3% (S1, 12.9%; S2, 18.4%).

Mangrove forests can produce relatively large amounts of organic matter through the conversion of leaf litter into detritus [64], that are later exported to nearby habitats [95–97]. Therefore, the proximity of the mangrove stands to shallow water seagrass meadows will most likely contribute to the higher biodiversity and, particularly, higher density observed within seagrasses. The populations of suspension-feeders, such as *Barbatia foliata,* which was dominant in the seagrass meadow (S1), supports the idea of higher availability of organic suspended particulate matter derived from, among others, nearby mangrove canopies and this higher availability will also support more resident organisms [68, 99]. Despite the high temporal variability observed in all habitats, highlighted by the dissimilarity indices, seagrass habitats showed a comparatively higher stability, with the lowest values of inter-annual variability, similar to previous studies in temperate areas [8, 98]. These habitats also supported the highest number of resident species (i.e. those present in over 85% of the sampling periods). At the lagoon entrance, the exclusive presence of *Schizaster gibberulus*, a sea urchin previously associated with the near shore coastal biotope in the region [16], suggests that the unvegetated area may be located along a corridor connecting offshore and lagoon communities, with patterns likely dependent on the hydrodynamic processes [99]. Its position between the lagoon and the open coastal water may also explain the high number of species observed (121), with a large proportion being exclusively associated with this habitat (33.9%). It is worth noting that given the generally low density observed in the Red Sea [16, 100], future studies will require to increase the replication across multiple spatial scale to fully understand the dynamics of benthic macroinvertebrates under low nutrient, high temperature, and high salinity conditions. Therefore, conclusions related to abundance and diversity should be interpreted with caution.

The present findings reinforce the need for an integrated understanding of shallow-water habitats from a seascape perspective, in opposition to a fragmented analysis of the isolated habitats [21, 101, 102]. Whereas the latter may be relevant when looking at particular species, the contribution of each habitat to the dynamics of the whole macrobenthic assemblages is relevant and should not be disregarded by managers when aiming for marine biodiversity conservation. Indeed, in tropical regions, seagrass beds and mangroves have been reported as key nursery areas for several reef fishes such as parrotfishes (Labridae, Scarini), grunts (Haemulidae) and snappers (Lutjanidae) [103–106] that rely on the macrobenthos as food resources. Large-scale migrations (over 30 km) by juvenile snappers, between inshore nursery habitats and reefs in the central Red Sea have been reported [22]. Also, mangrove forests have been linked to enhanced biomass and biodiversity of coral reef fishes [18, 21, 104, 107, 108]. Sustained connectivity of the habitats may enhance the resilience of coral populations to recover after disturbance [107]. Therefore, disturbing the corridors connecting coral reefs with other inshore habitats may even have consequences for reef conservation at a local scale.

## Conclusion

Overall, the present study confirmed a decreasing gradient in the total number of species and number of exclusive species towards the mangrove habitats. It also supports the role of seagrass habitat complexity in promoting diversity and density of organisms. Nevertheless, high and stable seagrass cover does not necessarily result in the highest biodiversity levels. But the presence of these plants plays an essential role in the biodiversity of coastal lagoons. Seagrass habitats in contrast to mangrove forests and the unvegetated area show lower inter-annual variability and higher number of resident species, suggesting more stable communities.

Current findings highlight habitat-structured patterns and persistent patchiness evidenced by a limited number of overlapping species (dominance of habitat specialists over generalists) within the seascape. This is particularly relevant considering the proximity of the analysed habitats but may result from the low dominance levels compared to temperate regions [92, 98, 109]. Nevertheless, 22.8% of the gamma diversity was represented by taxa spanning between subtidal and intertidal habitats. Hence, holistic, i.e. interconnected seascape management approaches, rather than those focusing on single habitats should be prioritized to protect biodiversity and fisheries [22, 110, 111].

## Methods

### Study area and sampling design

The present study was carried out in the Al Qadimah lagoon (22° 22′ 39.3″ N, 39° 07′ 47.2″ E) located in the central region of the Saudi Arabian Red Sea (Fig. 1a). This shallow lagoon (average depth 2.19 m) has an approximate area of 14 km^2^ and is not impacted by direct anthropogenic disturbances typical of other coastal lagoons (e.g. freshwater or sewage discharges, fisheries, habitat destruction from coastal development). It is, however, situated between two urbanized areas, which are increasing in size (King Abdullah University of Science and Technology, 7000 inhabitants; King Abdullah Economic City, currently 5000 inhabitants but it is expected to reach 50,000 in the near future) but that are not directly connected with the lagoon. Hence, it offers a rare opportunity to study the natural roles of environmental drivers in shaping macrobenthic communities inhabiting such critical wetlands.

Scattered along the extent of its margins, well-developed mangrove stands of *Avicennia marina* are observed. The bottom of the lagoon, particularly in the inner areas is characterized by more or less fragmented seagrass meadows. To depths of approximately 50 cm, *Cymodocea rotundata* is the dominant species with smaller patches of *Cymodocea serrulata* also being present. Below this depth, seagrass meadows are mainly characterized by mono-specific stands of *Enhalus acoroides* down to 2 m depth. Towards the sea, unvegetated bottoms with either sponges mixed with coral rubble or sand progressively replace seagrass meadows.

In the Red Sea, there are two marked seasons (Fig. 1b), winter (November–April) and summer (May–October). In order to investigate inter-annual and seasonal changes in macrobenthic patterns, samples were collected in two different periods in winter (January; March) and summer (June; September) of 2014 and 2015. Five permanent soft-sediment habitats typical of tropical coastal lagoons were selected: 1. upper mangrove area (M1); 2. deeper mangrove area (M2); 3. shallow seagrass meadow (S1, mix meadows of *Cymodocea serrulata* interspaced with *Cymodocea rotundata*; relatively high cover all year round); 4. deeper seagrass meadow (S2, monospecific stands of *Enhalus acoroides* with high variability in the vegetation cover throughout the study period); and 5. unvegetated soft-sediments (Fig. 1a). The unvegetated sandy substrate was located between 8 and 10 m depth. Due to the widespread distribution of seagrasses, mangroves and in order to minimize the direct influence of those habitats on the colonization patterns of unvegetated areas, the site was located at the entrance of the lagoon.

### Sampling strategy

At each habitat and sampling period, conductivity, temperature, and depth (CTD) casts were carried out with a multiparameter probe (OCEAN SEVEN 316 Plus and 305 Plus). The CTD casts also recorded oxygen saturation in the water column. Water samples for the analysis of chlorophyll *a* (chl *a*) were collected using a Niskin bottle at each station (2 L per station). Sediment samples were collected using a 0.1 m^2^ Van Veen grab in the seagrass meadows and the unvegetated area (subtidal stations), whereas in the mangrove habitats (intertidal), samples were collected using hand corers (3 × 10 cm i.d. making one replicate; total area per replicate ~ 0.024 m^2^). In 2014, two replicates at each site and sampling date were taken for the study of macrobenthic communities, with additional samples being collected for the study of environmental variables (grain particle size distributions and organic matter content). In 2015, the same approach was followed increasing the number of replicates for the study of macrobenthic communities to three. Macrobenthic samples were sieved through 1 mm mesh screens and preserved in 96% ethanol.

### Laboratory analyses

In order to estimate the primary production in the sampling area, the concentration of chl *a* was quantified by fluorescence using the EPA method 445.0 [112]. Water samples were filtered using GF/F filters as soon as we arrived at the laboratory. The filters were then preserved at -80 °C until extraction of the pigments. 10 ml of 90% acetone were used for each extract and left for 24 h in cold and dark conditions to minimize degradation. The procedure was undertaken in low light conditions to minimize degradation. A Turner Trilogy^®^ fluorometer (Turner Designs) was used to quantify the chl *a* content using an acidic module. The degradation of the chlorophyll *a* to phaeophytin was accomplished by acidifying the sample with 60 µl of 0.1 N HCl.

Sediment samples were sorted after all the vegetation associated with sediment was removed. Organisms were whenever possible identified to the species level. Vegetation biomass (seagrass leaves, roots, and mangrove material) was quantified per replicate.

Grain particle-size distribution was quantified after initial wet sieving of the samples (63 μm mesh) to separate the silt and clay fraction from sandy fractions and gravel. The retained fractions were dried at 80 °C for 24–48 h. The dried sandy and gravel sample was then mechanically sieved by using a column of sieves to separate the sandy fractions and the gravel as follows: < 63 μm, silt–clay; 63–125 μm, fine sand; 250-500 μm, medium sand; 1000–2000 μm, coarse sand;  > 2000 μm, gravel.

The organic content of the sediments was determined by loss on ignition (LOI). Sediments were dried for 24–48 h at 60 °C and then the samples were placed in the muffle furnace at 450 °C for 4 h. After cooling in a desiccator for 30 min, samples were weighed and the LOI was calculated using the following equation [113]:

$$\text{LOI} = {(\text{W}_\text{i}}-{\text{W}_\text{f}})/{\text{W}_\text{i}} \times 100$$

where: LOI = Organic Matter content (%), W_*i*_ = Initial weight of the dried sediment subsample; W_*f*_ = Final weight after ignition.

### Data analysis

#### General patterns

Macrobenthic patterns were analysed through a combination of univariate and multivariate techniques. Several univariate metrics were calculated including the total number of taxa (S, species richness), density (ind. m^−2^), and Shannon–Wiener (H′). Considering the different sampling methods, and the dependency of species richness on sample size [114], estimates of species diversity were also calculated and compared with S. The nonparametric species richness estimators used: Chao 1, Jacknife 1 order and Bootstrap all follow an asymptotic approach to estimate the number of undetected species richness. These estimators are commonly used in ecological studies because they are simple, intuitive, relatively easy to use and perform reasonably well [115]. The biased corrected form of Chao 1 estimator [114, 116] uses the number of singletons and doubletons to estimate the lower bound of species richness. The first order Jacknife estimator [117] assumes that the number of species that are missed equals the ones that were seen once (singletons). The Bootstrap estimator is based on the assumption that if the same data is resampled with replacement the number of missing species after resampling will be similar to those missed originally [117]. All estimators were calculated using the open source software R [118] using function “specpool” from “vegan” package [119]. Abundance data was used for the calculations of all estimators. In order to have a balanced number of replicates, the analyses were conducted for two replicates, with those collected in 2015 being randomly selected. Preliminary analysis showed that the same general patterns in composition and alpha-diversity were obtained for 2014 and 2015 datasets.

To visualize multivariate patterns of abundance in macrobenthic communities within the seascape, non-metric multidimensional scaling (nMDS) was applied based on the Bray–Curtis dissimilarities. Given the differences among habitats for some dominant species, when comparing habitats (i.e. full dataset), Bray–Curtis dissimilarities matrices were calculated using untransformed abundance data. Separate nMDS plots were generated for each one of the sites for a better visualization of the temporal variability. These analyses were also based on untransformed data. Within each site, significant variability in the multivariate patterns over time was analysed initially according to a three-factor design (Year; Season; Date, nested within Season) using Permutational Multivariate Analysis of Variance (PERMANOVA). As the factor “Date” was found not significant, and to increase the power of the analysis, a two-factor PERMANOVA was applied. Whenever significant differences in the interaction term were detected (i.e. Year × Season), pair-wise tests were conducted.

#### Connectedness within the seascape and stability patterns over time

A preliminary investigation of the patterns of variability across the seascape was carried out to identify generalist versus specialist taxa, i.e. those that span across multiple habitats versus those that are particularly associated with a specific habitat, respectively. We aimed to characterize the main differences in the community patterns in terms of shared and exclusive species that could determine the cause of the connectivity across the lagoon. This analysis was conducted based on the whole dataset, disregarding the seasonal and annual changes, as our main question was related to the constancy of spatial changes in different habitats.

Finally, we analysed the frequency of occurrence of species in each habitat during the study period. Species were classified based on Habitat Preference Trait as follows: (i) resident, present in over 85% of the sampling dates (i.e. eight events); (ii) frequent, observed between 50% and 85% of the dates; (iii) occasional, presence registered in between 25% and 50% of the sampling occasions; (iv) rare, observed in less than or equal to 25% of the sampling dates; (v) seasonal, only observed in one season but in both years.

Community stability was also examined over the sampling period within each habitat based on the indices Bray–Curtis (community structure) and Jaccard (presence/absence; composition). Within each habitat, variability between all pairwise comparisons among terms of interest (e.g. within and between seasons; within and between years) was analysed. We established that low levels of similarity are related to high variability in the macrobenthic communities over time, whereas high similarity is indicative of more stable communities.

#### Relationships between environmental variables and assemblage structure

Distance-based redundancy analysis (dbRDA) was used to assess the relationship between each environmental variable and the variation in the community structure (given by the direction and length of vectors for each variable). The variables used for the analysis were salinity, temperature, depth, grain size fractions, organic matter content (% LOI), and chl *a*. Three of the variables were transformed to reduce skewness, namely the fines and coarse sand fractions of the sediment (square root) and organic matter content (natural log). Marginal tests are used to show the significance of each variable individually to the model and sequential tests show the best subset of explanatory variables that explain the biological patterns.

## Data Availability

The datasets used and/or analysed during the current study are available from the corresponding author on reasonable request.

## Abbreviations

C/N: Carbon to Nitrogen ratio
Chl *a*: Chlorophyll *a*
CTD: Conductivity, temperature, and depth casts
dbRDA: Distance-based redundancy analysis
*H’*: Shannon–Wiener
LOI: Loss on ignition
M1: Upper mangrove area
M2: Deeper mangrove area
MDS: Non-metric multidimensional scaling
OTUs: Operational taxonomic units
PERMANOVA: Permutational Multivariate Analysis of Variance
S1: Shallow seagrass meadow
S2: Deeper seagrass meadow
Unv: Unvegetated soft-sediments
